# Degradation of Antibiotics in Wastewater: New Advances in Cavitational Treatments

**DOI:** 10.3390/molecules26030617

**Published:** 2021-01-25

**Authors:** Emanuela Calcio Gaudino, Erica Canova, Pengyun Liu, Zhilin Wu, Giancarlo Cravotto

**Affiliations:** 1 Dipartimento di Scienza e Tecnologia del Farmaco, University of Turin, Via P. Giuria 9, 10125 Turin, Italy; emanuela.calcio@unito.it (E.C.G.); erica.canova@unito.it (E.C.); pengyun.liu@unito.it (P.L.); zhilin.wu@unito.it (Z.W.); 2 Huvepharma Italia Srl, Via Roberto Lepetit, 142, 12075 Garessio (CN), Italy; 3 Institute for Translational Medicine and Biotechnology, First Moscow State Medical University (Sechenov), 8 Trubetskaya ul, Moscow 119048, Russia

**Keywords:** ultrasound, hydrodynamic cavitation, antibiotic residues, wastewater treatment, sono-photochemical processes, oxidative degradations

## Abstract

Over the past few decades, antibiotics have been considered emerging pollutants due to their persistence in aquatic ecosystems. Even at low concentrations, these pollutants contribute to the phenomenon of antibiotic resistance, while their degradation is still a longstanding challenge for wastewater treatment. In the present literature survey, we review the recent advances in synergistic techniques for antibiotic degradation in wastewater that combine either ultrasound (US) or hydrodynamic cavitation (HC) and oxidative, photo-catalytic, and enzymatic strategies. The degradation of sulfadiazine by HC/persulfate (PS)/H_2_O_2_/α-Fe_2_O_3_, US/PS/Fe^0^, and sono-photocatalysis with MgO@CNT nanocomposites processes; the degradation of tetracycline by US/H_2_O_2_/Fe_3_O_4_, US/O_3_/goethite, and HC/photocatalysis with TiO_2_ (P25) sono-photocatalysis with rGO/CdWO_4_ protocols; and the degradation of amoxicillin by US/Oxone^®^/Co^2+^ are discussed. In general, a higher efficiency of antibiotics removal and a faster structure degradation rate are reported under US or HC conditions as compared with the corresponding silent conditions. However, the removal of ciprofloxacin hydrochloride reached only 51% with US-assisted laccase-catalysis, though it was higher than those using US or enzymatic treatment alone. Moreover, a COD removal higher than 85% in several effluents of the pharmaceutical industry (500–7500 mg/L COD) was achieved by the US/O_3_/CuO process.

## 1. Introduction

Concern about wastewater treatment has recently increased tremendously due to the presence of pollutants from various industries. The adverse effects of pharmaceutical contaminants in water have driven attention towards their treatment. In particular, antibiotics are commonly detected in wastewater [1]. As many of these substances are recalcitrant to the conventional primary and secondary processes applied in municipal wastewater treatment plants [2], they can end up in natural waters. Pollution from antibiotics in the biosphere induces bacterial selection and causes microbial resistance [3]. Antibiotic-resistant bacteria can be transferred to humans, thereby increasing the risk of illnesses and diminishing our ability to treat them. Consequently, suitable processes to effectively degrade antibiotics in wastewater must be developed. Besides adsorption strategies [4] and hydrothermal treatments [5], advanced oxidation processes (AOPs) are the most commonly employed and cost-effective technologies for the complete degradation of persistent organic contaminants, such as antibiotics, in wastewater [6,7]. AOPs have been defined as water-treatment processes that are based on the in situ generation of strong oxygen-based reactive species, which promote the destruction of the target pollutant in water via mineralization [8,9]. The most common AOPs are based on the in situ generation of ^•^OH radicals by means of a range in chemical, sonochemical, photochemical, and electrochemical reactions. Of these reactions, the oldest chemical AOP, in which a mixture of a soluble iron (II) salt and H_2_O_2_—together known as Fenton’s reagent—is used to destroy and degrade persistent organic pollutants (including antibiotic moieties) in wastewater, is particularly worthy of mention [10]. However, it is possible to greatly boost the oxidation efficiency of this method by simultaneously irradiating the treated wastewater sample with ultraviolet (UV) light (photo-Fenton method) or sunlight (solar photo-Fenton method) [11]. Moreover, other photochemical processes, such as heterogeneous photocatalysis, using TiO_2_ suspensions, and ozonolysis (O_3_ + UV irradiation), have been documented [12,13]. Another fascinating improvement in the field of wastewater treatment is the combination of electrochemical reactions with Fenton’s reagent to create so-called electrochemical advanced oxidation processes (EAOPs) [14]. Since they use a clean reagent, electrons, EAOPs are an emergent and environmentally friendly technique for wastewater treatment that can either reduce or almost completely avoid the use of chemical reagents. In particular, hydroxyl radicals can either be produced directly via the oxidation of water on a high O_2_ evolution overvoltage anode (anodic oxidation) or indirectly in a bulk solution by means of a Fenton’s reagent that is electrochemically generated from electrode reactions. A novel electro-Fenton process was also developed for wastewater treatment using a modified divided electrolytic system in which H_2_O_2_ was generated in situ from electro-generated H_2_ and O_2_ in the presence of Pd/C catalyst [15].

Other AOPs include electrical plasma discharge and radiolysis, which have recently been applied in wastewater treatments. The former leads to extreme physical conditions that generate shockwaves, radicals (such as ·H, ·O, and ·OH), and UV light, which exert a direct pyrolysis effect on the contaminants [16,17]. The main features of radio-technology include simultaneously accomplishing both oxidative and reductive processes for the degradation of organic pollutants in water [18].

Several studies on AOP were also conducted to assess the feasibility of using ionizing radiation, in the form of gamma (γ) rays or electron beams (e^-^), to remove persistent contaminants in wastewater, showing technically and economically promising results [19]. Finally, new approaches to intensify chemical oxygen demand (COD) removal in wastewater when applied with a generalized Fenton reaction were assessed using constant magnetic field [20,21].

**Figure 1:**
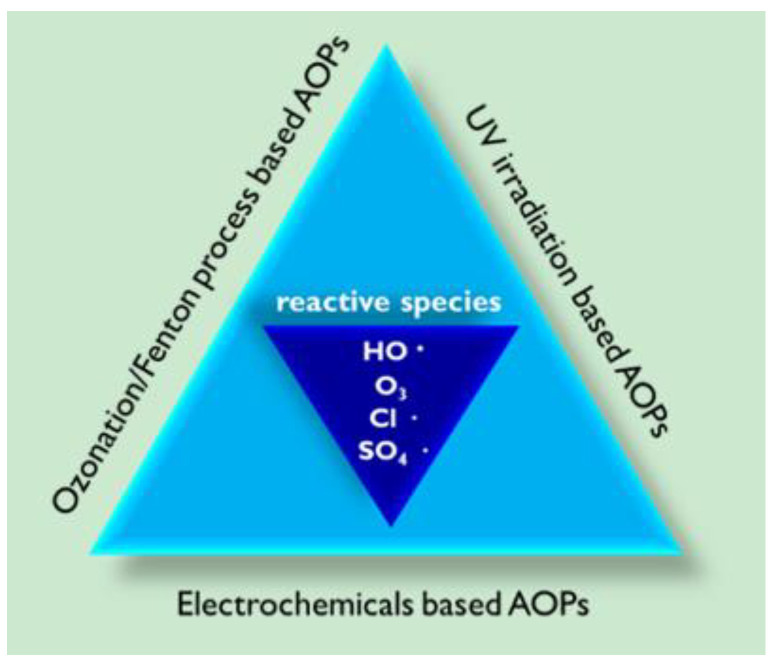
Integrated AOP classification and the reactive species involved.

However, some of the limitations of individual AOPs include their ineffectiveness against a wide range of bio-resistant organic contaminants, a poor mineralization degree (i.e., incomplete degradation), and the generation of toxic by-products [22]. In this context, the development of hybrid AOPs which combine two or more individual AOPs (Figure 1) has the potential to dramatically boost the degradation efficiency of organic contaminants in wastewater treatments by reducing treatment times and increasing mineralization yields [23,24,25].

Cavitation is the phenomena of the formation, growth, and violent implosion of vapor bubbles in a liquid medium [26,27]. The introduction of ultrasonic waves in aqueous solutions is another way to produce hydroxyl radicals. This process induces the formation, growth to a critical size, and violent collapse of micro-bubbles, creating singular temperature (5000 K) and pressure (1000 atm) conditions [28]. Under these conditions, dissolved oxygen and water are dissociated to produce hydroxyl radicals (Equations (1)–(4)).

(1) $$H_{2}O\;+\;)))\to \;\cdot H\;+\;\cdot \mathrm{OH}$$

(2) $$O_{2}\;+\;)))\;\to 2\;\cdot O$$

(3) $$H_{2}O\;+\;\cdot O\;\to \;2\;\cdot \mathrm{OH}$$

(4) $$O_{2}\;+\cdot H\;\to \cdot \;O\;+\;\cdot \;\mathrm{OH}$$

Ultrasonically and hydrodynamically induced cavitation are the two simplest methods. The first has been extensively studied as a means to degrade organic pollutants in water [29], whereas ultrasound (US) irradiation is not economically feasible for industrial plants due to its ineffective distribution of the cavitational activity on a large scale and the inefficient transfer of electric power into the liquid [30]. Hydrodynamic cavitation (HC), which can overcome the problems of ultrasonic cavitation, is a valid alternative technique [31]. As liquid passes through constrictions, such as orifices and venturi, the pressure at the throat, or *vena contracta*, falls below the vapor pressure of the liquid and the liquid flashes, generating a number of bubbles that will subsequently collapse when the pressure recovers downstream of the constriction [32]. Bubble collapse during cavitation generates localized transient hot spots with high temperatures and pressures which induce the cleavage of water and volatile pollutant molecules to yield free radicals [33], just like under acoustic cavitation. Despite many obvious differences in appearance, on a small scale the principles that govern hydrodynamic bubbles and acoustic bubbles are basically the same. However, as the use of acoustic cavitation in water and wastewater treatment (cleaning) is a well-known procedure, the use of hydrodynamic cavitation, alone or in combination with other strategies, has only recently been suggested and employed [34]. Despite the scarcity of examples, hydrodynamic cavitation has emerged as the best processing technology for wastewater treatment, considering its scalability and efficiency [35]. The combination of heterogeneous sonocatalysis and sonophotocatalysis is among the AOP hybrid techniques that have recently attracted the most attention, as it is a non-destructive and highly efficient method to address the toxic environmental impact of wastewater that contains hazardous pollutants [36]. The synergistic effect of sonocatalysis and photocatalysis converts organic contaminants and their intermediates to H_2_O and CO_2_ by generating huge numbers of robust radicals, such as hydroxyl (^•^OH), hydroperoxyl (HO_2_^•^), and superoxide (O_2_^•−^), and thus the overall oxidation process is enhanced. These highly oxidant species with a low selectivity are able to oxidize nearly all contaminant molecules via either hydrogen atom abstraction or hydroxyl group addition to the double bonds, leading to the formation of compounds with a lower molecular weight intermediate [37,38]. The application of these oxidation processes seems to be a promising alternative for the elimination of antibiotics from water [39].

The present review describes the recent synergistic approaches that combine acoustic and hydrodynamic cavitation and different chemical and physical methods for the degradation of antibiotics in water and wastewater. In several cases, conventional degradation methods have been compared with cavitational protocols. An overview of the works published between 2010 and 2020 on this topic is presented in this review in Table 1.

## 2. Oxidative Protocols Coupled with Cavitational Treatments

### 2.1. HC/PS/α-Fe_2_O_3_ and H_2_O_2_/Fe_3_O_4_

Persulfate (PS)-based oxidative degradation permits higher solubility and stability than OH radical-based AOP. Moreover, S_2_O_8_^2−^ can be activated through various means, such as thermal, UV, US, and transition metal ions, to generate persulfate radicals (SO_4_^•−^) [54]. Roy K. et al., (2019) [40] have demonstrated the increased effectiveness of a heterogeneous Fenton/PS system in the oxidative degradation of SDZ when combined with HC treatments. For this purpose, they synthesized two new solid Fenton catalysts (α-Fe_2_O_3_ and Fe_3_O_4_) that were obtained through hydrothermal (the former) and solvothermal (the latter) techniques in nanoparticle form.

**Figure 2:**
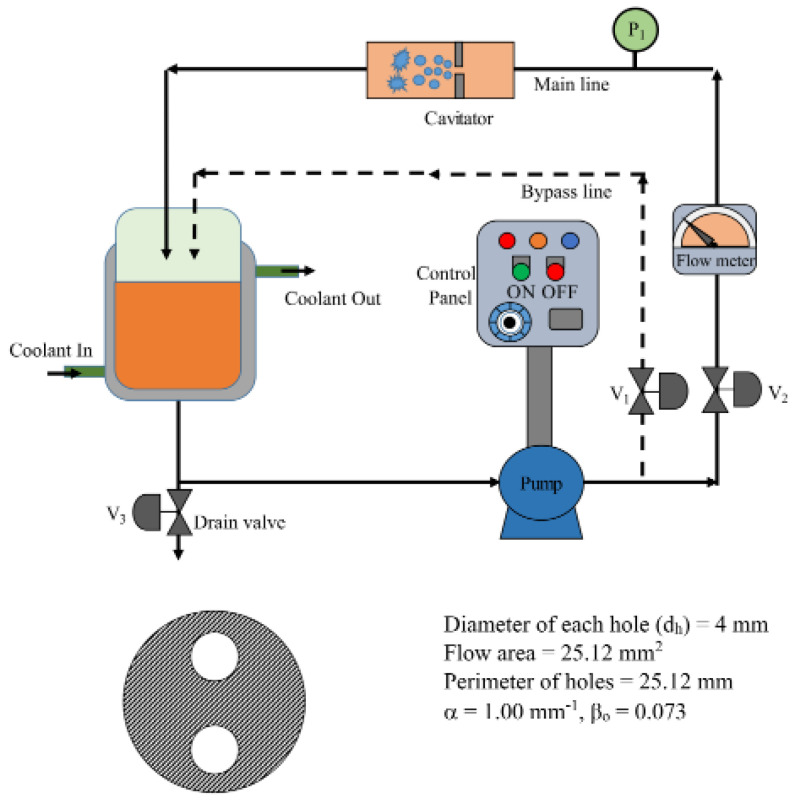
Schematic representation of the HC reactor for the oxidative degradation of SDZ. Reprinted with permission from [40].

The HC reactor used was a closed-loop circuit composed of a feed tank, main line, bypass line, and reciprocating pump (1.1 kW), as reported in Figure 2.

The rationale for the use of nanoparticle forms of Fenton catalysts was their higher surface area together with the higher stability of Fe^2+^ under prolonged treatment compared to zerovalent iron. SDZ degradation was performed both under HC treatment alone and under the combination of heterogeneous Fenton (α-Fe_2_O_3_ and Fe_3_O_4_) and PS treatment. The influence of four operating parameters was studied: antibiotic initial concentration; inlet pressure of cavitating flow; pH; and the type of heterogeneous Fenton catalyst used (at optimum loading). The highest SDZ degradation (81.0%) was achieved using α-Fe_2_O_3_ under the following conditions: SDZ initial concentration = 20 ppm; pH = 4; inlet pressure = 10 atm; Na_2_S_2_O_8_ = 348.5 mg/L; H_2_O_2_ = 0.95 mL/L; and α-Fe_2_O_3_ catalyst (at loading of 181.8 mg/L). 

An analysis of the experimental degradation profiles of SDZ showed that there was a high concentration of Fe^2+^ in the reaction mixture due to the intense turbulence and shear generated in the cavitating flow. The catalytic activity of α-Fe_2_O_3_ nanoparticles was proven to be efficient; in fact, the kinetic model analysis indicated the best match between the experimental and simulated profiles ([Fe^2+^]/[H_2_O_2_]) as an initial ratio = 4. This was possible thanks to the intense turbulence and shear generated in the cavitating flow in the HC reactor, which gave a high rate of mass transfer at the surface of the α-Fe_2_O_3_ particles. 

### 2.2. US/H_2_O_2_/Fe_3_O_4_

**Figure 3:**
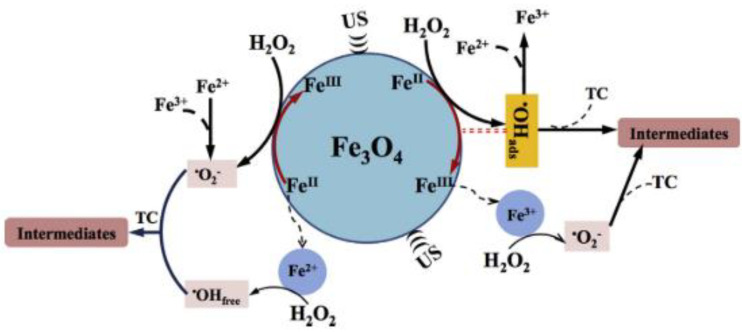
Proposed mechanism for the US/H_2_O_2_/Fe_3_O_4_ system. Reprinted with permission from [43].

Hydroxyl-radical-chemistry-based AOPs have been successfully employed for TC degradation [55]. The activation of hydrogen peroxide (H_2_O_2_) over heterogeneous Fe-bearing minerals has recently been demonstrated to be highly efficient in treating organic pollutants over a wide pH range. In this context, Hou L. et al., (2016) [43] recently reported the degradation of tetracycline (TC) over a magnetite (Fe_3_O_4_) catalyst using a coupled US/heterogeneous Fenton process (US/H_2_O_2_/Fe_3_O_4_). Magnetite was selected as the iron oxide for two key reasons: (1) it contains Fe^2+^ sites, which can react with H_2_O_2_ to generate ^•^OH; (2) it has low solubility in the reaction medium. Furthermore, compared with other transition metal oxides, Fe_3_O_4_ is cheap, non-toxic, and easily recyclable, making it extremely attractive for wastewater remediation processes. Experiments with several different scavengers indicated that ^•^OH_ads_ plays a key role in the TC degradation process. Nevertheless, the combined application of US technology was crucial to enhancing the production of radical species and overcoming the poisoning of the heterogeneous catalyst by degradation process intermediates. The sonocatalytic process gave the best degradation efficiency, in terms of TC, when coupled with H_2_O_2_: a 93.6% TC removal was achieved in 60 min of treatment (KQ-100 KDB ultrasonic generator, 100 W, 20 kHz,), with a total organic carbon (TOC) removal rate of 31.8%, whereas only 72.2% TC degradation was achieved with the H_2_O_2_/Fe_3_O_4_ system. Energy consumption was also compared and, although the sonocatalytic process provided fewer energy savings (1319.8 kWh/(m^3^ order)) than the corresponding silent system (872.7 kWh/(m^3^ order)), the higher TC removal efficiency and higher reaction rate achieved under US irradiation made US/H_2_O_2_/Fe_3_O_4_ the system of choice (Figure 3).

Several process parameters were screened for their impact on the TC degradation rate (ultrasonic power, reaction temperature, pH, initial TC concentration, Fe_3_O_4_ loading, and H_2_O_2_ concentration), and the results achieved can be summarized as follows:

- an increase in TC removal efficiency was only observed when the US power was above 80 W;
- an increase in pH corresponds to an increase in the degradation rate because the Fe^2+^ on the catalyst can more easily activate H_2_O_2_ under acidic conditions to produce more hydroxyl radicals on the catalyst surface;
- an increase in [H_2_O_2_] corresponds to an increase in the degradation rate (a concentration of 150 mmol/L corresponded to 93.6% TC removal), but a further increase in the H_2_O_2_ concentration to 250 mmol/L barely improved the TC removal (93.7%);
- an increase in the Fe_3_O_4_ loading (from 0.5 to 1.0 g/L) resulted in an increase in the removal efficiency, but a further increase was not seen when the loading exceeded 1.0 g/L;
- modifying temperature (within a range of 20–50 °C) increased the rate of degradation linearly with the temperature increase.

Moreover, the Fe_2_O_3_ catalyst exhibited a good structural stability under US irradiation and a limited loss in performance upon three reuses. These findings suggest that US/H_2_O_2_/Fe_3_O_4_ is a promising method to enhance the degradation of TC and other toxic and non-biodegradable pollutants.

### 2.3. US/PS/Fe^0^ System

**Figure 4:**
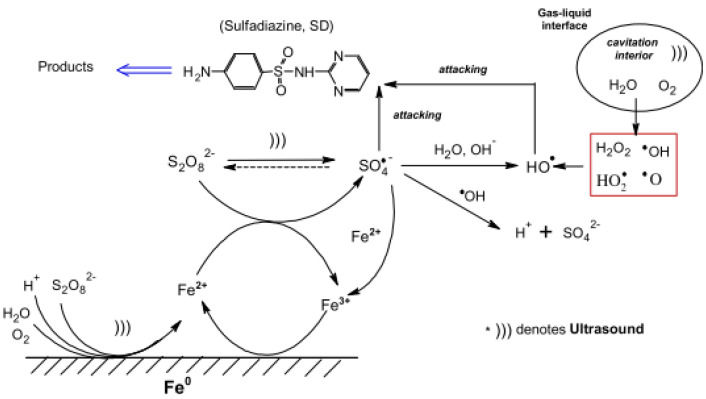
Scheme of the reaction mechanism in the US/PS/Fe^0^ system and the promotional role of US. Reprinted with permission from [41].

Zou X et al., (2014) [41] reported a combined US/PS/Fe^0^ system for efficient SDZ degradation. Zerovalent iron, a cheap and non-toxic catalyst, was exploited as an alternative activator for SO_4_^•−^ production under US treatment. Moreover, Fe^0^ could recycle Fe^3+^ on its surface and reduce the precipitation of iron hydroxides during the reaction [56]. A VCX750 ultrasonic horn processor working at 20 kHz was adopted for this purpose and the US input powers were 20, 40, 90, and 140 W, respectively. The SDZ aqueous solution (10–20 mg/L) was placed in a jacket-glass reactor and a thermostatted bath was used to keep the reaction temperature constant (10–50 °C). Three different combined systems, US/Fe^0^, PS/Fe^0^, and US/PS/Fe^0^, were adopted for SDZ degradation, and it was demonstrated that the three-component system (US/PS/Fe^0^) provided a significant degradation efficiency (>95% after 1 h treatment). US effectively accelerated the Fe^0^ corrosion and the bulk radical reactions, activating PS to the sulfate radical. Moreover, the main parameters, such as initial SDZ concentration, [Fe^0^]:[PS] dosage ratio, pH, US input power, and reaction temperature, were investigated for SDZ degradation under US/Fe^0^/PS treatment. The principle results were as follows:

- a gradual increase (from 52.7% to 90.9%) in the SDZ degradation efficiency was achieved with an increase in the PS concentration;
- acidic circumstances favored SDZ degradation;
- with a high initial concentration of SDZ for the production of radicals (SO_4_^•−^ and ^•^OH) [57], the degradation efficiency increased with increasing US input power;
- a higher temperature was favorable for degradation;
- the decomposition rate of PS in the US/PS/Fe^0^ system was faster than in the US/PS and PS/Fe^0^ analogues and followed pseudo first-order kinetics with a *k*_obs_ (SDZ) value of 3.4 ± 0.20 h^−1^. The reaction mechanism for the US/PS/Fe^0^ system is indicated in Figure 4.

HPLC–ESI-MS was used to reveal the SDZ degradation pathways, and five main compounds were identified (*m*/*z* 279, 230, 214, 185, and 120, respectively). The author concluded that, under US/PS/Fe^0^ synergistic treatment, sulfate radical oxidation was the main SDZ degradation pathway. The attack on the amine group in the benzene ring would be the first step, and then the C–N bonds in the heterocyclic ring were cleaved. Another hypothesized degradation pathway was the direct cleavage of the S–N bond.

### 2.4. US/Oxone^®^/Co^2+^

Shengnan S. et al., (2012) [42] investigated AMX degradation via sulfate radicals in aqueous solution under US irradiation. Preliminary tests were performed using Oxone^®^ (2KHSO_5_·KHSO_4_·K_2_SO_4_) as the source of the strongly oxidant peroxymonosulfate ion (HSO_5_^−^) and sulfate radicals (SO_4_^−^). Since Anipsitakis et al., (2003) [58] indicated that Co^2+^ is the best transition metal catalyst for Oxone^®^ activation (Equations (5) and (6), Oxone^®^ was exploited by the authors for AMX oxidative degradation both alone and in combination with Co(NO_3_)_2_ under US irradiation at 20 kHz (by means of a probe device).

(5) $${\mathrm{Co}}^{2+}+{\;\mathrm{HSO}}_{5}^{-}\;\to \;{\mathrm{Co}}^{3+}\;+\;{\mathrm{SO}}_{4}\cdot^{-}\;+\;{\mathrm{OH}}^{-}$$

(6) $${\mathrm{Co}}^{3+}+{\;\mathrm{HSO}}_{5}^{-}\;\to \;{\mathrm{Co}}^{2+}\;+\;{\mathrm{SO}}_{5}\cdot^{-}\;+\;H^{+}$$

The adopted concentrations of AMX, Oxone^®^, and cobalt solution were 0.095, 5, and 0.025 mmol/L, respectively. 

**Figure 5:**
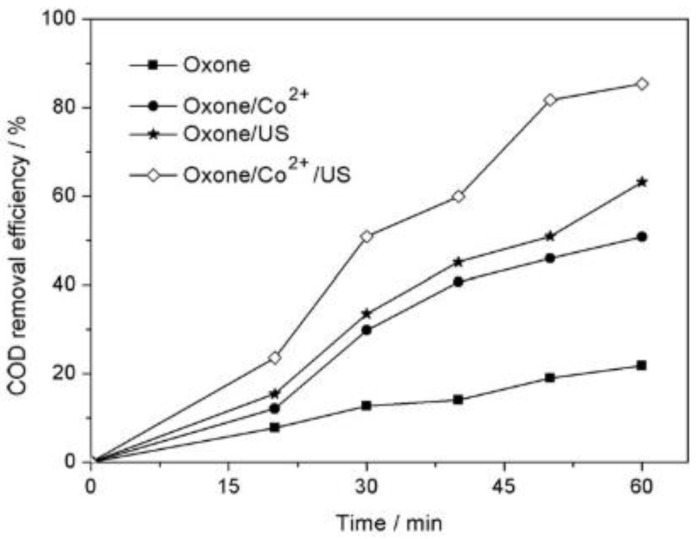
Effect of different treatment methods at room temperature and near-neutral pH in terms of the COD removal efficiency. Experimental conditions: [AMX] = 0.095 mmol/L, [Oxone^®^] = 5 mmol/L [Co^2+^] = 0.025 mmol/L, T = 24 °C, US = 20 KHz, 200 W. Reprinted with permission from [42].

The preliminary oxidative tests clearly identified US as the best and most efficient method for treating AMX wastewater when coupled with cobalt-activated Oxone^®^. The synergistic effect of US/Oxone^®^/Co^2+^ was documented by the increased COD removal efficiency: for 60 min of oxidative treatment under Oxone^®^ (22%) < Oxone^®^/Co^2+^ (51%) < US/Oxone^®^ (63%) < US/Oxone^®^/Co^2+^ (85%) at near neutral pH and room temperature (Figure 5). 

Moreover, an analysis of the effect of some operating parameters (temperature, US power, concentration of Oxone^®^ and cobalt ion, initial AMX concentration) on the degradation of AMX gave the following results:

- the COD removal efficiency increased with increasing temperature, in particular from 24 to 40 °C;
- increasing the US power flow from 100 to 300 W enhanced the COD removal efficiency from 58.4% to 93.5%, while from 400 to 500 W the COD removal efficiency decreased from 81.1% to 74.2%;
- increasing [Oxone^®^] enhanced the COD removal efficiency from 72.9% to 98.5%;
- increasing [Co^2+^] enhanced the COD removal efficiency from 57.2% (0.005 mmol/L) to 98.7% (0.045 mmol/L);
- increasing [AMX] from 0.072 to 0.119 mmol/L improved the COD removal efficiency from 76% to 93.4%, while, at [AMX] above 0.119 mmol/L, the COD decreased.

This study demonstrated that the mechanism of AMX degradation obeys first-order kinetics. However, the US-assisted process can significantly decrease the energy barrier necessary for the oxidation of AMX.

### 2.5. US/O_3_/Goethite Process

**Figure 6:**
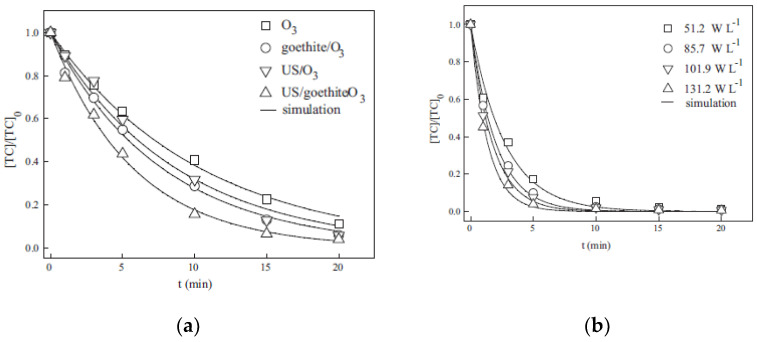
(**a**) Comparative performance for the different oxidation conditions in TC removal; (**b**) effect of US power density on TC removal. Reprinted with permission from [44].

The ozonation process has been receiving increasing attention in the treatment of pharmaceutical wastewater due to its ability to oxidize complex organic compounds into simpler and more easily biodegraded compounds [59]. However, its widespread application has been limited by low use and the limited oxidizing power of ozone. Therefore, ozone is usually combined with homogeneous and heterogeneous catalysts or other activating technologies (including UV and US) to enhance its oxidation power and improve its use. Moreover, catalytic ozonation boosts the degradation of recalcitrant pollutants due to its ability to enhance ozone decomposition [60]). In this context, Wang et al., (2011) [44] reported a comparative study of TC degradation by a combination of O_3_ and heterogeneous goethite catalysts using a specific rectangular air-lift US reactor under different ozonation conditions. As shown in Figure 6a, the removal of TC by the above processes (ozonation, catalytic ozonation, US/O_3_ process, and US/O_3_/goethite process) follows apparent pseudo first-order kinetics according to the following rate equation:

(7) $$-\;\frac{d\;\left[\mathrm{TC}\right]}{d\;t}\;=k\left[\mathrm{TC}\right]$$

where [TC] is the TC concentration at time t and k is the pseudo first-order degradation rate constant.

Only a 0.096 min^−1^ rate constant was achieved by ozonation, while the rate constant increased to 0.115 min^−1^ when ozonation was combined with ultrasonic irradiation. These effects can be considered to be reasons for the enhancement of the mass transfer of ozone from the gaseous phase to the aqueous phase. In the meantime, the decomposition of ozone thermolytically :

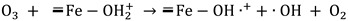
(8)

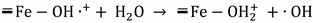
(9)

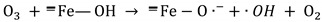
(10)

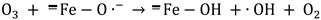
(11)

The generated hydroxyl radicals would then either attack TC at the surface of goethite, or oxidize TC in solution when they diffuse into the bulk liquid phase. This resulted in a higher degradation rate being achieved by catalytic ozonation than by ozonation alone. When both ultrasonic irradiation and the goethite catalyst were introduced into the ozonation system, the TC removal rate reached 0.174 min^−1^, which was nearly twice as high as that for ozonation alone, and higher than that in the catalytic ozonation and US/O_3_ systems. Moreover, the TC removal rate soared with increasing US power density (Figure 6b).

As discussed above, TC oxidation by ozone was improved when US irradiation and a catalyst is combined with ozonation. The active sites of the catalyst would be gradually occupied by the oxidation intermediates during the catalytic ozonation process, which results in catalyst deactivation. In the presence of ultrasonic irradiation, the available active sites can be maintained due to the cleaning action of US. In the meantime, US may cause catalyst fragmentation and increase the surface area as well as the number of active sites, while the turbulent effect of cavitation can enhance the mass transfer of the reactants and products to and from the catalyst. On the other hand, the presence of a solid catalyst may increase the formation of cavitation. Therefore, compared to ozonation, catalytic ozonation and US/O_3_, much higher degradation rates were achieved by the US/O_3_/goethite system. When oxygen was bubbled into the reactor instead of a mixture of oxygen and ozone, little TC removal was observed (data not shown). This illustrates that the amount of free radicals generated was negligible, and, consequently, little TC was degraded during the US/goethite process as the hydroxyl radical and singlet oxygen are considered to be the main oxide species during the ozonation process [61]. The addition of tert-butyl alcohol (TBA) as a stronger hydroxyl radical scavenger or sodium azide as a singlet oxygen scavenger to the US/O_3_/goethite system was then investigated. The presence of these free radical scavengers inhibited the TC removal rate, indicating that both the direct molecular ozone reaction and the indirect radical reaction were involved in the US/O_3_/goethite system. TC was oxidized quickly by ozone, free radicals, or both via 1,3-dipolar cycloaddition and electrophilic reactions to form immediate products. After three consecutive cycles, the TC removal rate and the particle size distribution of goethite change insignificantly, and the dissolved iron concentration was very low, indicating the stability of this catalyst in the US/O_3_/goethite system.

### 2.6. US/O_3_/CuO

**Figure 7:**
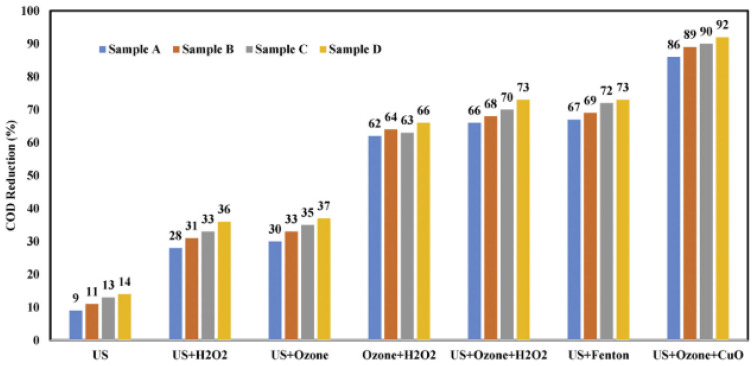
Comparison of different approaches for the COD reduction of various PIE samples. Reprinted with permission from [45].

**Figure 8:**
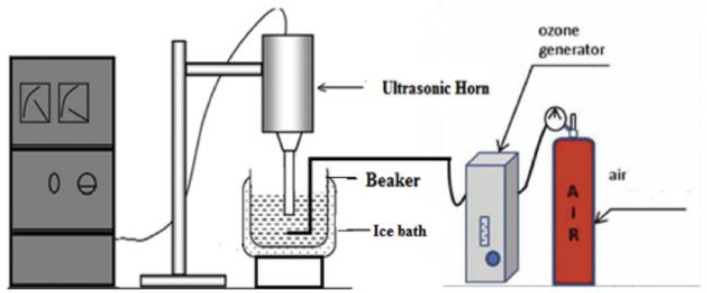
Schematic representation of experimental setup for degradation with US/ozone combination. Reprinted with permission from [45].

Chandak S. et al., (2020) [45] recently investigated the US degradation of different pharmaceutical industry effluents (PIE) by exploiting the combined use of different oxidative agents. In particular, under controlled US operating conditions using an ultrasonic horn device set at 22 kHz, 250 W, for 120 min at room temperature, the addition of hydrogen peroxide (various loadings of 1:1, 1:5, 1:10), Ozone (flow rate of 400 mg/h), CuO catalyst (fixed loading as 0.3 g/L) and Fenton’s reagent (FeSO_4_:H_2_O_2_ ratio of 3:5) was evaluated in order to establish the best combined degradation approach. Different PIE samples with initial CODs of 7500 (A), 6500 (B), 3000 (C), and 500 (D) ppm were selected as references from different processing stages, Figure 7. All of the experiments (only US; US/H_2_O_2_; US/O_3_; O_3_/H_2_O_2_; US/Fenton’s reagent; US/O_3_/H_2_O_2_; US/O_3_/H_2_O_2_; US/O_3_/CuO) were carried out at ambient temperature and pressure, and the samples were analyzed by high-resolution liquid chromatography mass spectroscopy (HR-LCMS). The authors confirm that US alone was not very effective at degrading pharmaceuticals, while US coupled with other AOPs can be efficiently used for the treatment of PIE with a significant increase in COD reduction. Ozone was identified as the best oxidant, and the best results were reported for the US/O_3_/CuO combined degradation approach (Figure 7), which enabled a percentage of COD decrease that was higher than 85% for all PIE samples. The equipment used for the combined US/O_3_/CuO is reported in Figure 8. It is important to emphasize that the addition of CuO was crucial for PIE degradation under US. In fact, the simple US/O_3_ combined approach gave only 37% COD reduction. Finally, after an HR-LCMS analysis of treated PIE samples, it was possible to confirm that the intermediate products formed during US treatment were non-toxic compounds.

## 3. Photocatalytic Strategies Coupled with Cavitational Treatments

Several studies on the photocatalytic degradation of antibiotics and their possible degradation pathway have been documented in the literature [62,63]. For example, Hu et al., (2016) [64] recently designed a composite material made of graphene, TiO_2_, and zeolite (ZSM-5) (identified as GTZ) that is suitable for the photocatalytic degradation of oxytetracycline (OTC) under visible light. The produced three-component GTZ, with a 1:8:1 mixing ratio, showed the best photocatalytic activity for OTC degradation (with a reaction rate constant of 4 × 10^−2^ min^−1^). In fact, the rate was better than those achievable over pure TiO_2_, graphene-TiO_2_ and the TiO_2_-ZSM-5 bi-composite. Mineralization with GTZ was monitored using a TOC analyzer and the reported results showed that, under optimal operating conditions (pH 7; 25 °C), the complete degradation (about 100%) of OTC was achieved in 180 min. However, some organic substances were still in the solution as only 78% TOC was removed after 240 min of radiation.

However, traditional photocatalytic degradation techniques are affected by mass-transfer limitations and gradual catalyst deactivation via the accumulation of by-products (or contaminants) on the catalyst surface. These might be significant obstacles for any large-scale application of conventional photocatalytic oxidation in real wastewater treatment. To solve these problems, many researchers have been attempting to introduce cavitation into photocatalytic reaction systems to improve the light utilization efficiency of suspended TiO_2_ [65,66]. 

### 3.1. HC/Photocatalysis with TiO_2_ (P25)

**Figure 9:**
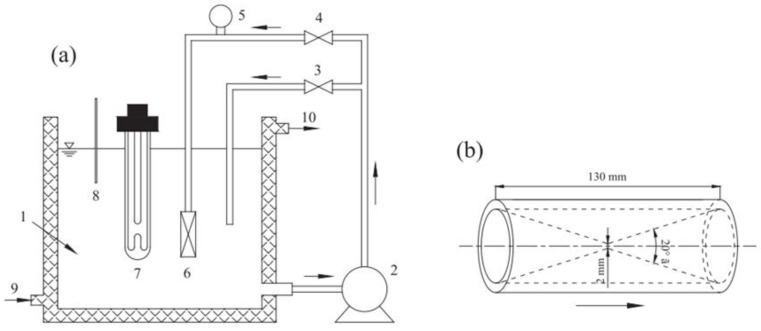
(**a**) Experimental setup for the HC and photocatalytic experiments on TC degradation. The venturi tube and straight pipe with the same length and inlet diameter were alternated, and the UV254 lamp was turned on as needed to conduct the individual or combined processes. (1) Reactor; (2) centrifugal pump; (3 and 4) throttle valves; (5) pressure gauge; (6) venturi tube or straight pipe; (7) UV254 lamp; (8) thermometer; (9) cooling water inlet; (10) cooling water outlet. (**b**) geometry of the venturi tube. Reprinted with permission from [46].

**Figure 10:**
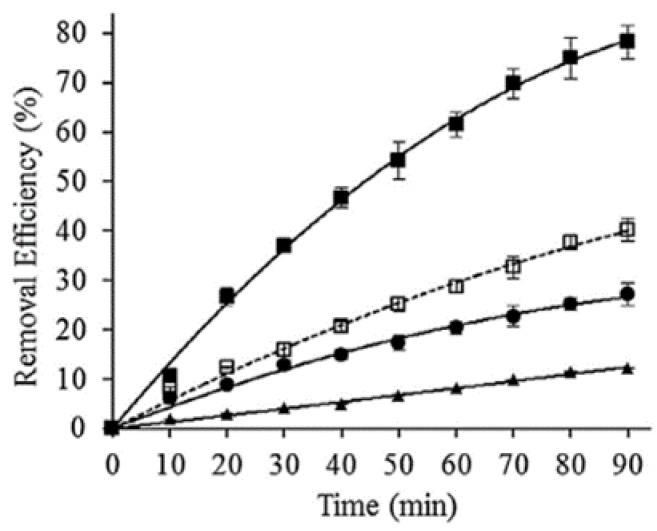
Degradation of 30 mg/L tetracycline in a 4.0 L solution following 90 min of (N) hydrodynamic cavitation alone, (d) photocatalysis under circulation agitation, 100 mg/L TiO_2_, and (j) simultaneous photocatalysis and hydrodynamic cavitation, 100 mg/L TiO_2_. h is the simple linear sum of N and d (initial pH was natural 4.2; no matrix component was added). Reprinted with permission from [46].

Although HC is a promising and green technology for the degradation and even mineralization of water contaminants, reports of hybrid combinations of the HC and photocatalytic degradation of pharmaceuticals are rare [67]. In one of the few documented examples, Wang et al., (2017) [46] recently combined photocatalysis and HC (by means of a venturi tube) to degrade TC in an aqueous solution using TiO_2_ (P25). For this purpose, TC degradation experiments were performed in a jacketed cylindrical glass reactor (capacity 5.0 L) and a jet flow loop (Figure 9). UV254 irradiation was provided by a 9 W mercury lamp, which was hosted in a 30 mm diameter quartz tube and placed vertically inside the reactor. The aqueous solution in the reactor was circulated at 30 °C through the jet-flow loop, which was driven by a high-pressure, self-priming stainless-steel pump. A venturi tube (made of Pyrex glass) acted as the producer of cavitation. The degradation of TC via individual HC, photocatalysis, and a combination of photocatalysis with HC was evaluated first (Figure 10). The combined approach was proven to be more effective than the individual ones (Figure 10), as it led to a TC removal efficiency of 78.2% after 90 min treatment, which is about two times higher than the sum of those reported for the individual HC and photocatalytic approaches (12.2% and 28.1%, respectively). Undoubtedly, a synergistic effect occurred during the simultaneous application of HC and photocatalysis. In particular, the ability of HC to prevent the agglomeration of catalyst particles and the ability to constantly refresh its surface, via the action of micro-jets and shockwaves generated by cavitation, played a pivotal role in the catalyst photoactivity throughout the combined process. In addition, the radical species created by HC, though in relatively small amounts compared to acoustic cavitation, can also support the photocatalytic reaction. Moreover, many factors, such as initial tetracycline concentration, solution pH, and the presence of inorganic anions, were investigated. The degradation of TC by individual HC, photocatalysis and a combination of the two processes was observed to apparently follow the first-order rate law under a variety of initial contaminant concentrations. Using a fixed TiO_2_ dose of 100 mg/L, with an increase in the initial TC concentration from 10 to 80 mg/L, the rate constant of photocatalysis coupled with HC (k_1_) decreased from 21.9 to 7.8 × 10^−3^ min^−1^. This is much higher than the value of HC alone (k_2_) and of photocatalysis with circulation agitation (k_3_) under the same treatment conditions. A Synergistic Coefficient (SC) was used to evaluate the synergic effect of photocatalysis coupled with HC. This parameter demonstrated that the combination of photocatalysis and HC enhanced the degradation rate of TC by a factor of 1.5–3.7 compared to the simple linear sum of the degradation rates under independent processes, and that this increases with higher initial TC concentrations. Moreover, the authors demonstrated how the degradation rate of TC was pH-dependent and favored under alkaline conditions. The presence of HCO_3_^−^ improved the TC degradation rate in the combined process, whereas sulfate and chloride had a minor effect. HCO_3_^−^ (pH 8.3) gave very rapid degradation in the first 20 min of the treatment. This behavior was caused by the reaction between bicarbonate ions and hydroxyl radicals, generating the carbonate radical, which tends to selectively react with electron-rich compounds.

The combination of photocatalysis and HC had a synergistic effect on the degradation of TC. Their degradation pathway was also hypothesized by the author to follow a hydroxylation and deamination step in addition to the possible destruction of (other) functional groups on the TC structure. 

### 3.2. Sonophotocatalysis with rGO/CdWO_4_

**Figure 11:**
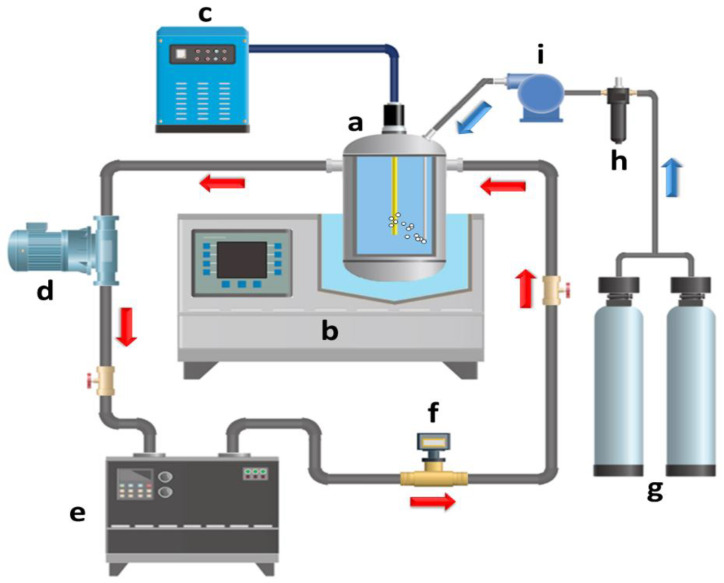
Schematic of the sonophotoreactor setup: (a) photoreactor cell, (b) US bath, (c) UV irradiation control panel, (d) circulation pump, (e) water bath, (f) flow-meter, (g) oxygen gas, (h) gas filter, and (i) oxygen diffuser pump. Reprinted with permission from [47].

Photocatalysts commonly play an imperative role in sonophotocatalytic processes. To date, various photocatalysts have been reported for the decomposition of organic pollutants in aqueous matrices, but the lack of efficient photoactivity under solar-light, the high recombination rate of photo-induced electron-hole (e_CB_^−^-h_VB_^+^) pairs, and low resistance to photo-corrosion has limited their practicality in environmental applications. Due to their relatively narrow band gap and excellent photocatalytic activity under solar-light irradiation, tungsten-based photocatalysts, such as Na_2_W_4_O_13_, Bi_2_W_2_O_9_, CdWO_4_, ZnWO_4_, PbWO_4_, AgIn(WO_4_)_2_, and LiCr(WO_4_)_2_, have received significant attention over the past decade [68]. In particular, CdWO_4_, with its monoclinic wolframite structure, has generated great interest as a visible-light responsive photocatalyst (VLPHS) for heterogeneous catalytic systems [69]. Moreover, reduced graphene oxide (rGO), which has a two-dimensional structure with a one-atom thickness, is a suitable support to anchor VLPHS as a way to enhance the transfer and separation of photo-induced charge carriers due to its excellent charge-mobility (approximately 200,000 cm^2^ V^−1^ s ^−1^), large specific surface area, and high mechanical and thermal stability [70]. In this context, a hierarchically arranged flower-like rGO/CdWO_4_ VLPHS has been recently synthesized and tested as a robust solar-light-responsive photocatalyst for the US-assisted degradation of tetracycline (TC) by Ghoreishian et al., (2019) [47]. This is one of the first reported examples of a synergistic approach in which a VLPHS has been used to degrade TC via sonophotocatalysis using a binary rGO/CdWO_4_ composite under simulated visible-light irradiation (Figure 11). Studies of structural characterization confirmed that flower-like CdWO_4_ particles with a wolframite phase structure were successfully immobilized on the surface of the GO. Consequently, hierarchically arranged flower-like rGO/CdWO_4_ VLPHS were successfully synthesized via the facile photocatalytic reduction of GO/CdWO_4_, using a simple wet-chemical method without any calcinations. Furthermore, PEG-4000 was added to regulate the morphology of nanocrystalline CdWO_4_, and the VLPHS layers were coated onto Indium-Tin-Oxide, using the spin-coating method, and dried in an oven at 80 °C for 3 h. For the TC degradation tests, a certain amount of rGO/CdWO_4_ was first dispersed into 500 mL of a TC solution (pH-adjusted) and then stirred for 1h in the dark until the VLPHS and TC adsorption-desorption equilibrium was reached. In the second phase, irradiation and sonication were utilized, and this moment was taken as the initiation time of the sono-photo-degradation process.

**Figure 12:**
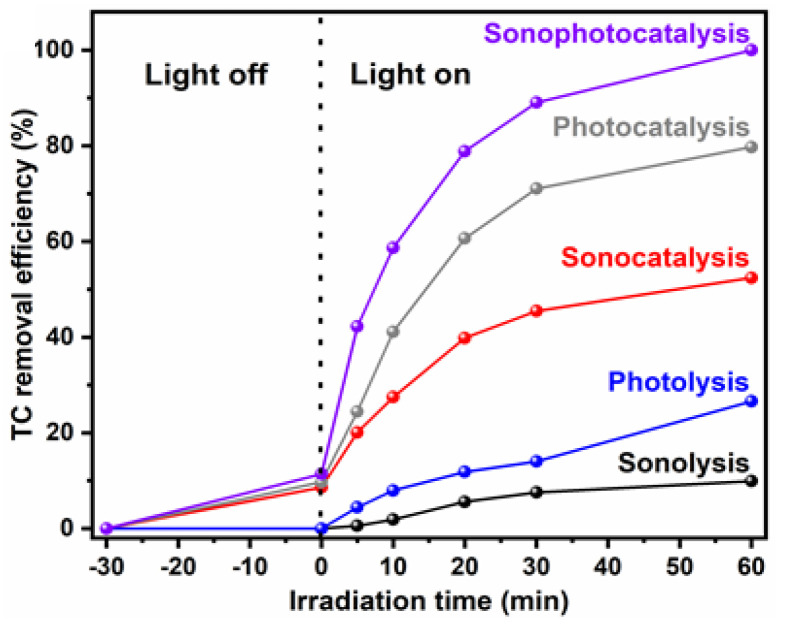
Comparison of the TC removal efficiency as a function of irradiation time. Reprinted with permission from [47].

The TC degradation efficiencies of rGO/CdWO_4_, GO/CdWO_4_, and CdWO_4_ were compared under optimized sonophotocatalytic process parameters (pH 5.7 [TC]_0_ = 13.54 mg/L, treatment time = 60 min, and catalyst dosage = 0.216 g). Complete sonophotocatalytic degradation was described for rGO/CdWO_4_ after 1 h of treatment at 25 °C, whereas GO/CdWO_4_ and CdWO_4_ gave only 39.64% and 13.46% degradation efficiencies over the same period. Thus, it is clear that TC degradation efficiency can be dramatically increased by the presence of rGO/CdWO_4_, compared to GO/CdWO_4_ and pure CdWO_4_. This improvement may be due principally to the superior specific surface areas, which provide more adsorption sites and active catalytic reaction centers, and the lower *E*_g_ value of rGO/CdWO_4_, which enhances the light absorption efficiency in the visible range. Consequently, this system (rGO/CdWO_4_) was very effective because it showed significant photoelectrochemical behavior, superior sonophotocatalytic activity, and a good mineralization efficiency. Furthermore, when the catalytic efficiency of rGO/CdWO_4_ is compared with that of commercial photocatalysts, rGO/CdWO_4_ has proven to be more active than ZnO (1.5 times) and TiO_2_ (3 times) for TC degradation under the same optimized sonophotocatalytic conditions. The higher catalytic activity of rGO/CdWO_4_ compared to other options was ascribed to rGO, which can absorb TC residuals in an aqueous solution and which acts as a charge acceptor, promoting the separation of photo-generated carriers via its π-π conjugated structure. Furthermore, rGO/CdWO_4_ has double the photocurrent (40 μA cm^−2^) of bare CdWO_4_. The stability and recyclability of rGO/CdWO_4_ were also evaluated under optimal conditions, and rGO/CdWO_4_ VLPHS showed a high stability in a four-cycle degradation process (240 min). In order to better understand the contribution of each individual and hybrid TC degradation processes, a comparative experiment into sonolysis (ultrasonication without rGO/CdWO_4_), photolysis (light irradiation without rGO/CdWO_4_), sonocatalysis (ultrasonication with rGO/CdWO_4_), photocatalysis, and sonophotocatalysis was conducted under the optimal conditions. As reported in Figure 12, after 60 min 100% TC was degraded by sonophotocatalysis (as previously reported), while sonolysis, photolysis, sonocatalysis, and photocatalysis exhibited relatively low removal efficiencies of 9.93, 26.6, 52.41, and 79.74%, respectively.

**Figure 13:**
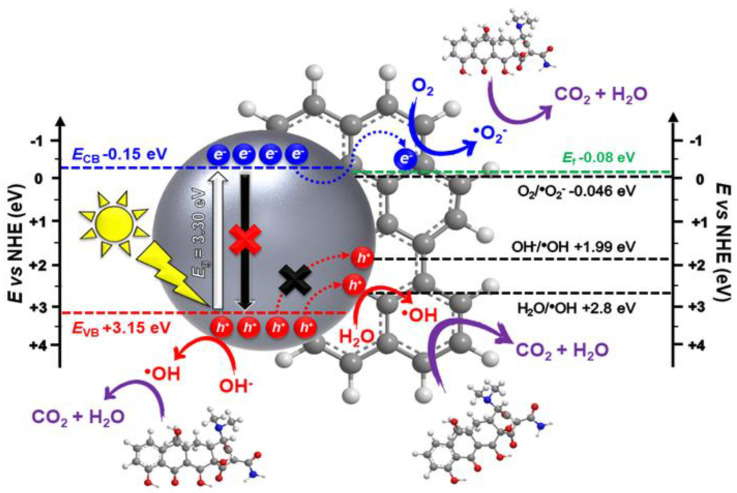
The possible mechanism of photocatalytic degradation of TC in the presence of rGO/CdWO_4_ under solar irradiation. Reprinted with permission from [47].

Concerning the kinetics of sonophotocatalytic processes, the analysis confirmed that TC degradation followed the Langmuir–Hinshelwood kinetic model, and the authors tentatively proposed the photocatalytic mechanism behind the degradation of TC by rGO/CdWO_4_ (Figure 13).

### 3.3. US/UV with MgO@CNT Nanocomposites

Heterogeneous hybrid systems have more recently emerged as effective approaches for the decontamination of aqueous solutions containing non-biodegradable compounds. In this context, Hayaty et al., (2020) [48] exploited the addition of carbon nanotubes (CNTs), as one of the most frequently applied types of surface modification, to enhance the catalytic activity of magnesium oxide (MgO) in the US-assisted photocatalytic degradation of SDZ. To the best of our knowledge, this is the first study to employ hexagonal MgO anchored onto CNTs as a heterogeneous catalyst in combination with UV and US waves (marked as MCs/UV/US) for the degradation of SDZ. The MgO@CNT nanocomposites (MCs) were synthesized using a US-assisted hydrothermal method and fully characterized by the XRD, FESEM, TEM, FTIR, EIS, BET, TGA, UV–vis DRS, EDAX, and EDS techniques. In particular, the FESEM and TEM analyses revealed that CNTs were well-incorporated into the hexagonal MgO. The suitability of the MCs/UV/US system for SDZ decontamination was then evaluated as a function of initial SDZ concentration, pH solution, catalyst loading, UV intensity, US power, the addition of scavenging agents, and co-existing chemicals. MCs had a significant synergistic effect under both UV and US irradiation. Indeed, during the sonophotocatalytic process, the complete degradation of SDZ (45 mg/L) was achieved within 80 min at pH: 11.0; MCs: 0.9 g/L; UV intensity: 150 W and US power: 200 W. In particular, the SDZ degradation increased from 62.7 to 100%, upon increasing the intensity of light irradiation (from 50 to 150 W), and from 70.8% to 100% upon increasing the US power (from 100 to 250 W/m^2^).

Moreover, MCs exhibited high chemical stability and reusability after six consecutive cycles. Nevertheless, the addition of co-existing anions, especially Cl−, progressively decreased the efficiency of the MCs/UV/US hybrid system, probably because the anions can behave as free radical scavengers. Kinetic data confirmed that the degradation process fit a pseudo first-order kinetic model with a regression coefficient of 0.98. The MCs/UV/US process effectively quenched 89% and 96% of the TOC and COD in the pharmaceutical wastewater, respectively.

Trapping studies indicated that hydroxyl radicals and holes were responsible for the oxidation of organic chemicals during the reaction period (Equations (12)–(18)). Initially, US formed microbubbles in the solution, and visible light irradiation was then was created via US irradiation, which excited MCs to generate sono-induced charge carriers (e^−^/h^+^ pairs).

(12) $$H_{2}O\;+\;)))\;\to {\;\mathrm{HO}}^{\cdot }\;+\;H^{\cdot }$$

(13) $$H_{2}O\;+{\;H}^{\cdot }\;\to {\;\mathrm{HO}}^{\cdot }\;+\;H_{2}$$

(14) $${\mathrm{HO}}^{\cdot }\;+{\;\mathrm{HO}}^{\cdot }\;\to {\;H}_{2}O_{2}$$

(15) $${\mathrm{HO}}_{2}^{\cdot }\;+\;{\mathrm{HO}}_{2}^{\cdot }\;\;\to {\;H}_{2}O_{2}\;+\;O_{2}$$

(16) $$H_{2}O_{2}+\;{\mathrm{HO}}^{\cdot }\;\;\to {\;H}_{2}O\;\;+\;{\mathrm{HO}}_{2}^{\cdot }$$

(17) $$H_{2}O_{2}\;+\;\;)))\;\to \;2{\mathrm{HO}}^{\cdot }$$

(18) $$\mathrm{MCs}\;+\;)))\;\to {\;h}_{\mathrm{VB}}^{+}\;+\;e_{\mathrm{CB}}^{-}$$

**Figure 14:**
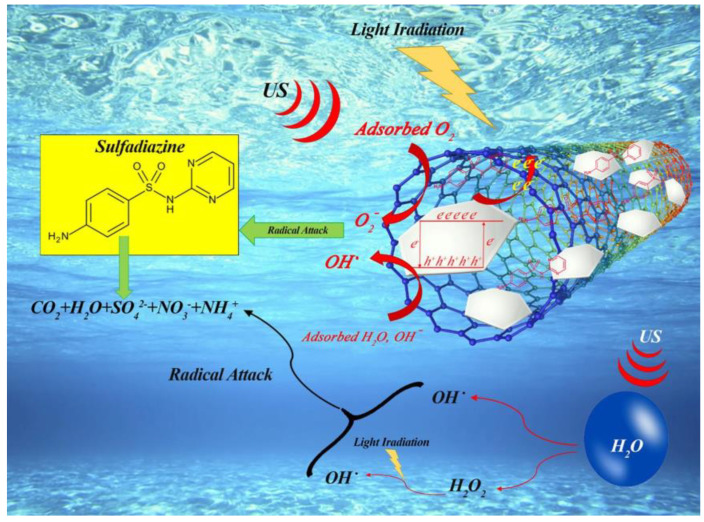
The possible mechanism for the detoxification of SDZ-containing wastewater in the MCs/UV/US system. Reprinted with permission from [48].

Moreover, a plausible pathway for the sonophotocatalytic decomposition of SDZ over MCs has been proposed (Figure 14). An SDZ degradation pattern was hypothesized in which negatively charged free radicals attacked the unstable C–N bonds and N–H bonds on the benzene ring and hydroxylation occurred at the pyrimidine ring, which was further hydroxylated.

Finally, lightweight compounds were converted into CO_2_, SO_4_^2−^, NH_4_^+^, NO_3_^−^, and water, and this was confirmed by the results of the TOC removal studies. In conclusion, this system showed an excellent mineralization performance which enhanced the biodegradability and decreased toxicity, while MCs demonstrated efficiency and high reusability. 

### 3.4. US-Assisted Photocatalyst Preparation 

Bismuth-based catalysts are another group of photocatalysts that can provide great activity under visible light, and this is due to the suitable band gap energy of these photocatalysts, which effectively impacts upon their absorption of light. To date, various bismuth-based photocatalysts, such as BiVO_4_, Bi_2_O_3_, Bi_2_MoO_6_, and Bi_2_WO_6_, have been exploited. However, bismuth oxyhalides (BiOX; X: Cl, Br, I) present better performance due to their unique tetragonal crystalline structure, in which each of the cationic layers of [Bi_2_O_2_] is surrounded by two layers of halogen anionic atoms to form layers of [X-Bi-O-Bi-X] [71]. Although BiOBr has unique photocatalytic properties [72], its light absorption and photocatalytic activity are low due to its band gap of 2.76 eV and the high recombination rate of electron hole pairs. The creation of solid solutions of BiOBr with other photocatalysts has recently been proposed to address this challenging issue. In this context, S. Fard et al., (2019) [49] newly described the one-pot US-assisted solvothermal synthesis of (BiOBr)_x_(Bi_7_O_9_I_3_)_1−x_ solid solutions with several x values (x = 0, 0.25, 0.5, 0.75, and 1) that can act as a new ball-flowerlike nanostructured photocatalyst for the active degradation of LVO under sunlight. The photocatalytic activity of the synthesized nano-photocatalysts was evaluated in the degradation of 50 mg/L LVO under simulated solar light irradiation over 120 min. According to the performed evaluations, the value of x has a great impact on the photocatalytic activity. The highest adsorption capacity of LVO in darkness as well as the highest LVO photo-catalytic degradation was recorded for the (BiOBr)_0.75_(Bi_7_O_9_I_3_)_0.25_-U solid solution that was fabricated under US irradiation (1 h) by means of an ultrasonic homogenizer (200 W power).

This result was ascribed to the high activation of (BiOBr)_0.75_(Bi_7_O_9_I_3_)_0.25_-U in the solar spectrum range due to the band gap at around 2.43 eV, more effective charge transportation and separations, a decrease in the recombination rate, a large specific surface area (125.3 m^2^/g), superior total pore volume (0.589 cm^3^/g), as well as uniform dispersion and improved nucleation thanks to the effects of US, which led to an intensification in the catalytic active sites. 

Moreover, all of the LVO photocatalytic degradation process parameters (such as photocatalyst loading, pollutant concentrations, and solution pH) were evaluated, and the results showed that the (BiOBr)_0.75_ (Bi_7_O_9_I_3_)_0.25_-U solid solution nano photocatalyst had good stability and good photocatalytic activity, even at the end of the fourth degradation cycle.

**Figure 15:**
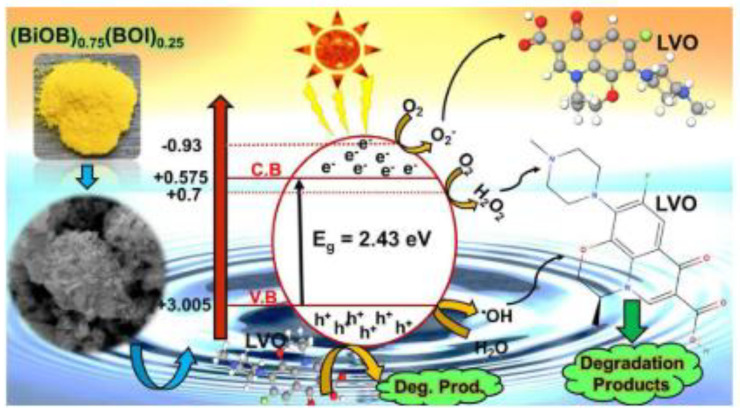
Reaction mechanism for sunlight photocatalytic degradation of antibiotic levofloxacin over (BOB)_0.75_(BOI)_0.25-U_ solid-solution nano-photocatalyst. Reprinted with permission from [49].

The reaction mechanism of LVO was also reported and can be seen, in scheme form, in Figure 15.

**Figure 16:**
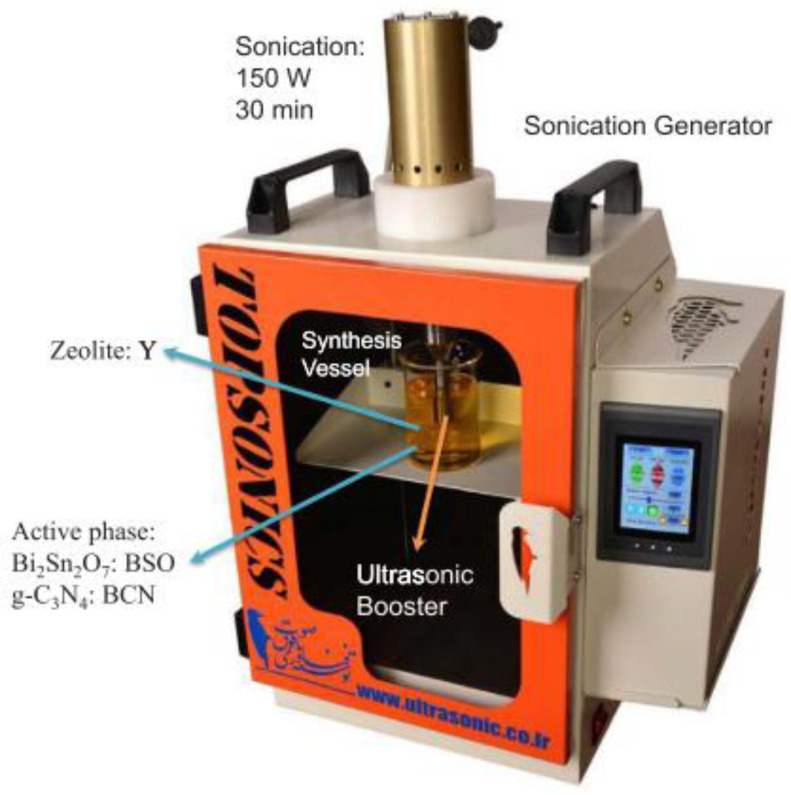
Experimental setup for the sono-dispersion of bulk Bi_2_Sn_2_O_7_ and g-C_3_N_4_ over zeolite Y. Reprinted with permission from [50].

S. Heidari et al., (2018) [50] reported a US-assisted dispersion of Bi_2_Sn_2_O_7_-C_3_N_4_ nano-photocatalyst (BSO-BCN) over various amounts of zeolite Y (Figure 16) as a means to enhance the solar-light photocatalytic degradation of tetracycline (TC) in aqueous solution. The photocatalytic activity of US-synthetized Bi_2_Sn_2_O_7_-C_3_N_4_ nano photocatalysts was evaluated for TC degradation (20 mg/L) under simulated solar-light irradiation for 90 min, and BSO-BCN/Y gave higher photocatalytic activity than BSO-BCN. The effect of different amounts zeolite Y (10, 20 and 30 wt%) was then evaluated, in terms of TC degradation efficiency, and BSO-BCN/Y-10% was found to be the best nano-photocatalyst with a degradation efficiency of about 80.4% at pH 6. Indeed, TC is neutral at this pH value and attracted to the photocatalyst surface without any repulsive force. 

**Figure 17:**
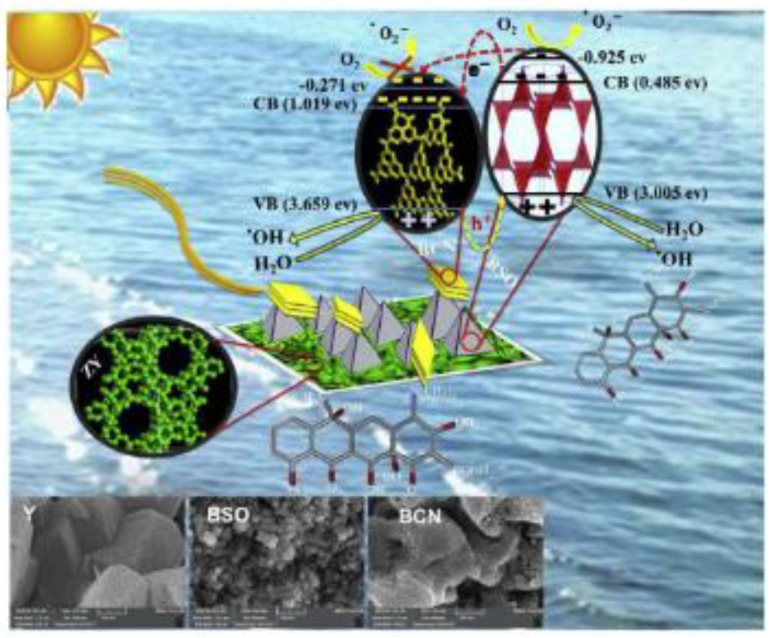
Reaction mechanism for the removal of tetracycline from water over the Bi_2_Sn_2_O_7_-C_3_N_4_/Y nano-photocatalyst. Reprinted with permission from [50].

On the other hand, the results of this study showed that the BSO-BCN/Y ternary composite has a higher photocatalytic activity than the BSOBCN binary composite, and this is attributed to (i) the uniform distribution of BSO-BCN over the support surface. However, the cation-exchange capacity of the zeolite causes the accumulation of pollutants on the photocatalyst surface and, as a result, more contact between the pollutant and active sites appears and the photocatalytic removal rate increases. Furthermore, photocatalyst conduction band electrons may be trapped in the zeolite structure. This phenomenon decreases the rate of recombination and boosts photocatalytic activity. The suggested mechanism for this photodegradation (Figure 17) involves the BSO-BCN/Y (10% wt) nano-photocatalyst being very active in producing active species.

## 4. Enzymatic Strategies Coupled with Cavitational Treatments

The rising scientific production in this area has recently shown that the degradation of particular pollutants is possible with the use of enzymes. While traditional degradation processes often require harsh conditions and the addition of chemicals, these drawbacks can be easily overcome by the concurrent application of enzymes with US for wastewater treatment. As has recently been documented by Rokhina et al. [73], enzyme stability and biocatalytic activity can be enhanced by US irradiation. Moreover, enzymes show many advantages compared to conventional biological treatments and/or chemical oxidation, and these include easy and simple process control, lower energy requirements, high tolerance over a wide pH range, salinity and temperature at low or high concentrations of pollutants, the absence of toxic effects, and the lack of generation of unforeseen products because of their high specificity. Laccase has been most scrutinized enzyme for pollutant degradations due to its broad substrate specificity and the use of oxygen, which is a non-limited electron acceptor. In particular, this enzyme is a multi-copper protein that catalyzes the oxidation of several aromatic and inorganic substances (particularly phenols) with the concomitant reduction of oxygen to water [74]. In this context, the first synergistic approach for Laccase-catalyzed CPFX hydrochloride degradation under US irradiation was documented by Sutar et al., (2015) [51]. A range of degradation parameters were screened (enzyme loading, temperature, agitation, US power, duty cycle, and frequency) in an ultrasonic thermostatic bath device (4.5 L capacity).

The results reveal that CPFX hydrochloride degradation under this US/enzymatic approach:

- increased (from 26% to 32%) with an increase in enzyme loading (from 0.015 to 0.02% (*w*/*v*)), but further increases over 0.02% (*w*/*v*) led to a reduction in degradation;
- increased (from 13% to 32%) with an increase in temperature (from 40 to 60 °C), but further temperature increases led to decreases in the degradation percentage;
- increased (from 13% to 51%) with an increase in US irradiation power (from 30 to 75 W), but further increases in the temperature led to decreases in the degradation percentage because temperature has a negative effect on cavitation. Further increases in the irradiation power, up to 100 W, decreased the degradation to 22%;
- decreased with an increase in US frequency (22 kHz and 40 kHz), and this is caused by smaller and less energetic bubble formation;
- increased (from 40% to 51%) with an increase in duty-cycle percentage (from 40 to 50%), but further increases over 60% led to reductions in degradation, which were caused by enzyme denaturation;
- increased (from 8% to 50%) with an increase in agitation speed (from 0 rpm to 200 rpm) due to improved mass transfer, but further increases over 300 rpm led to no further enhancements in degradation percentage.

The highest CPFX hydrochloride degradation of 51% was achieved at 0.02% (*w*/*v*) enzyme loading, 60 °C temperature, power input 75 W, 22 kHz frequency, 50% duty cycle, and agitation 200 rpm. Compared to the conventional method, this technique gave maximum CPFX degradation in less time, proving the synergistic effect of this coupled US/enzymatic approach. 

## 5. Multiple Synergistic Approaches in Comparison 

Tao Y. et al., (2018) [52] reported a novel wastewater degradation technique for antibiotic degradation that combines cavitating-jet impingement and multiple synergistic methods (UV/Fenton, analogous Fenton, and photocatalytic oxidation). Three different kinds of antibiotics—namely, AMX, doxycycline, and SDZ sodium—were chosen as model pollutants and individual applications of cavitating-jet impingement were initially applied to evaluate the effects of jet-impinging forms and nozzle-inlet pressure.

The effects of impingement were the promotion of antibiotic degradation, the enhanced generation of hydroxyl radicals, and weakened cavitating coalescing effects.

**Figure 18:**
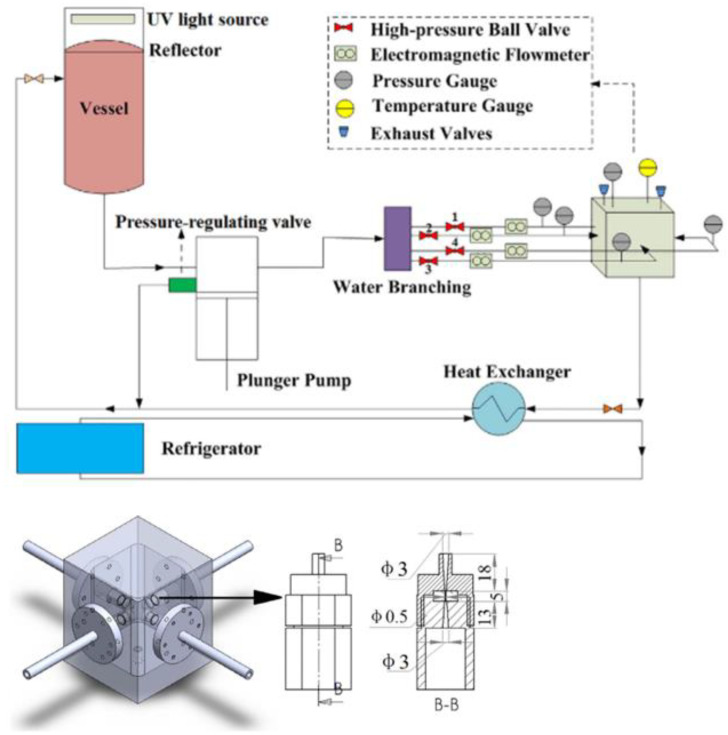
Schematic design of the experimental setup (**upper**) and reactor chamber (**bottom**). Reprinted with permission from [52].

The equipment used for antibiotic degradation was made up of three pipelines: the main line, the bypass line, and the cooling line, where the first contained a water vessel of 50 L, a high-pressure plunger pump of 5.5 kW, a water branching of four lines, a main reactor, and a heat exchanger (Figure 18). 

Perpendicular double cavitating-jet impingement has been proven to be the most effective impinging form, leading to a COD reduction of 30.04% with the impinging effect index of 1.22 at a jet-inlet pressure of 10 MPa. Moreover, increasing the nozzle inlet pressure enhances the impinging effect and COD reduction. The individual use of perpendicular double cavitating-jet impingement at a nozzle inlet pressure of 12 MPa induced the maximum COD reduction of 32.12%, with an impinging effect index of 1.46. On this basis, several process synergetic methods were successively adopted.

Among others, different loadings of H_2_O_2_ were added to the solution to investigate the effect on the degradation of the antibiotic. Increasing the H_2_O_2_ concentration significantly increased the COD reduction up to the maximum value at an H_2_O_2_ concentration of 500 mg/L. As the H_2_O_2_ concentration increases, the formation of hydroxyl radicals is enhanced due to the continuous dissociation of H_2_O_2_ under the cavitating and jet-impinging conditions, and thus the antibiotics degradation process is greatly promoted. However, when the H_2_O_2_ concentration is too high, a negative influence is generated due to the scavenging of hydroxyl radicals by H_2_O_2_ and the recombination reactions between hydroxyl radicals. Moreover, the presence of higher concentrations of H_2_O_2_ can also generate vaporous cavities, resulting in a lower cavitation intensity.

The effects of five factors on the combination of perpendicular double cavitating-jet impingement and a UV/Fenton process were investigated in orthogonal experiments, and the detected impact on COD was hydrogen peroxide concentration (A) > initial COD value (C) > pH solution (D) > temperature (E) > ferrous sulfate concentration (B). Under the effects of the combined cavitating-jet impingement and the UV/Fenton process, a COD reduction of 71.02% was achieved under the optimized conditions (initial H_2_O_2_ concentration of 500 mg/L, FeSO_4_ concentration of 10 mg/L, initial COD of 236 mg/L, pH of 2.7, and liquid temperature of 49 °C) and the synergetic index rose to 1.922.

After the analogous Fenton and photocatalytic oxidation methods were incorporated into the processing system, it was found that the use of CuSO_4_ and TiO_2_ can improve COD reduction under the optimum concentrations. Under the effects of the combined cavitating-jet impingement, UV/Fenton process, and analogous Fenton process, the maximum COD reduction increased to 76.02% and the synergetic index rose to 2.109. 

The combination of cavitating-jet impingement, UV/Fenton, analogous Fenton, and photocatalytic oxidation was found to be the optimal method for antibiotic wastewater treatment, with a COD reduction of 79.92% (maximum obtained COD), synergetic index of 2.125, and relatively good economic efficiency.

Serna-Galvis et al., (2016) [53] compared the potential degradation efficiencies of a problematic β-lactam antibiotic, oxacillin (OXA), in different oxidation approaches: TiO_2_ photocatalysis, sonochemistry, the photo-Fenton process, and electrochemistry (with a Ti/IrO_2_ anode in sodium chloride). Although each process performed OXA degradation successfully, significant differences were observed in the four oxidation approaches in terms of degradation pathways, by-product generation, and degree of mineralization. For example, photocatalysis and photo-Fenton mineralized, whereas sonochemistry and electrochemistry did not. Moreover, the main oxidation route was active chlorine attack on the electrochemical system, while, for the sonochemical and photo-Fenton treatments, the hydroxyl radical played the principal role. 

In TiO_2_ photocatalysis, the combined action of direct oxidation by the holes and the attack of hydroxyl radicals removed the antibiotic after 45 min, and about 90% mineralization was achieved after 135 min.

In the sonochemical system (275 kHz, 60 W), hydroxyl radicals degraded OXA at the interfacial zone and the pollutant was removed after 120 min of treatment. In the photo-Fenton process, the attack of hydroxyl radicals easily removed 100% of OXA and about 35% of the initial TOC after 27 min. However, only 9 min were necessary for the electrochemical oxidation to eliminate the OXA. Although OXA goes through the opening of the β-lactam nucleus and then undergoes oxidation at the thioether moiety and the breakdown of the secondary amide in all four oxidation processes, the intrinsic properties of each oxidation process lead to different OXA by-products. Moreover, because of the different nature of the applied processes, specific pathways to each were also found. For example, in TiO_2_ photocatalysis, the decarboxylation pathway was observed to occur through a possible photo-Kolbe reaction. The oxacillin isomerization pathway was promoted using the electrochemical process. Unlike the two AOPs (the photo-Fenton system and TiO_2_ photocatalysis), under sonochemical treatment initial hydroxylation was not observed on the aromatic ring. This comparative study demonstrated that the OXA-removal pathways and the efficiency of the primary and final degradation of the pollutant strongly depend on the oxidation process adopted.

## 6. Conclusions

Due to the recalcitrant nature of antibiotics residues, advanced chemical treatments—e.g., ozonation, UV/H_2_O_2_, UV/H_2_O_2_/Fe^2+^, and solar photo-Fenton processes—have recently emerged as alternative process in wastewater treatment. However, so far the relatively high cost of the equipment and the energy required to supply the processes are the major drawbacks that preclude their large-scale applicability. Cavitation-based technologies are attracting interest due to their cost effectiveness in operation, their minimization of toxic solvent usage, and their ability to obtain superior processed products compared to conventional methods. Higher removal efficiency and higher reaction rate of antibiotics are achieved under US or HC conditions as compared with the corresponding silent protocol. US and HC can strongly improve mass transfer, catalytic conversions, and the bulk radical generation, activating PS, oxone, and H_2_O_2_ to the sulfate, peroxymonosulfate, or hydroxyl radicals. The removal efficiencies of SDZ reached 81.0% and >95% in 60 min with the HC/PS/H_2_O_2_/α-Fe_2_O_3_ and US/PS/Fe^0^ process, respectively. Total AMX removals of 93.6% TC and 85% were achieved in 60 min with the US/H_2_O_2_/Fe_3_O_4_ and by US/Oxone^®^/Co^2+^ process, respectively. Due to the enhancement of O_3_ mass transfer and catalysis, the TC removal rate constant reached 0.174 min^−1^ with US/O_3_/goethite, which is nearly twice as high as that achieved using ozonation alone. Moreover, a higher than 85% COD removal for all pharmaceutical industry effluents (500–7500 mg/L COD) was achieved by the US/O_3_/CuO process. The TC removal efficiency after 90 min by HC/photocatalysis with TiO_2_ (P25) reached 78.2%, which is about two times higher than the sum of those reported for the individual HC and photocatalytic approaches (12.2% and 28.1%, respectively). Due to the superior specific surface area of rGO/CdWO_4_, the complete sono-photocatalytic degradation of TC was achieved in 60 min, whereas GO/CdWO_4_ and CdWO_4_ gave only 39.6% and 13.5% degradation efficiencies over the same period. Similarly, the complete degradation of SDZ was achieved in 80 min with a MgO@CNT nanocomposites treatment. 

Following the recent advances in cavitation technology with large-scale processing, hydrodynamic cavitation-based methods show easier applicability. Hybrid cavitational treatments may represent the solution of choice for the efficient degradation of antibiotics in wastewater.

## Figures and Tables

**Table 1:** Cavitational treatments for the degradation of antibiotics in wastewater: case studies reviewed.

Antibiotic	Process	Experimental Details	Ref.
**Oxidative Strategies Coupled with US/HC**			
Sulfadiazine (SDZ)	HC + Heterogeneous Fenton(α-Fe_2_O_3_)/Na_2_S_2_O_8_	Highest SDZ degradation = 81.0%; [SDZ]_0_ = 20 ppm; [H_2_O_2_]_0_ = 0.95 mL/L; [Na_2_S_2_O_8_] = 348.5 mg/L; pH = 4; Volume: 5 L (total); Time: 90 min (total); Inlet pressure = 10 atm.	[40]
SDZ	US + Fe^0^/K_2_S_2_O_8_	Highest SDZ degradation = 95.7%. [SDZ]_0_ = 10 and 20 mg/L; [K_2_S_2_O_8_]_0_ = 1.84 mM; Initial dosage ratio [K_2_S_2_O_8_]/[Fe^0^]: from 1:0.2 to 1:1, pH: 3.0–7.0; Volume: 400 mL Temperature: 10–50 °C; Reaction Time: 60 min US frequency and power: 20 kHz from 20 to 140 W.	[41]
Amoxicillin (AMX)	US + Oxone/Co^2+^	COD removal (Oxone/Co^2+^/US) = 85.0%; [AMX]_0_ = 0.095 mmol/L; [oxone]_0_ = 5 mmol/L; [Co^2+^] = 0.025 mmol/L; Neutral pH; Volume: 50 mL Reaction time: 60 min; US frequency and power: 20 kHz and 200 W.	[42]
Tetracycline (TC)	US+ Fe_3_O_4_/H_2_O_2_ over a magnetite catalyst	Highest TC degradation = 93.6%; [TC]_0_ = 100 mg/mL; [H_2_O_2_]_0_ = 150 mmol/L; [Fe_3_O_4_]_0_ = 1.0 g/L; pH_0_ = 3.7; Volume: 200 mL; Temperature: 22 ± 2 °C; Reaction time: 60 min; US frequency and power: 20 kHz and 80 W.	[43]
TC	US+ goethite/O_3_	TC degradation: 100.0%; [TC]_0_ = 100 mg/L; [goethite] = 0.5 g; [O_3_]_g_ = 13.8 mg/L; Q = 30 L/h; Volume: 200 mL Reaction time: 20 min; US frequency and power: 20 kHz and 250 W (max).	[44]
Pharmaceutical industry effluent (PIE)	US+ O_3_/CuO	Max. COD removal = 92.0%; Initial COD = 7500, 6500, 3000 and 500 ppm; O_3_ input amount = 400 mg/h; [CuO] = 0.3 g/L; Volume: 150 mL Room Temperature; Reaction time: 2 h; US frequency and power: 22 kHz and 250 W.	[45]
**Photocatalytic strategies coupled with US/HC**			
TC	HC+ Photocatalysis with TiO_2_ (P25)	Highest degradation = 78.2%; [TC]_0_ = 30 mg/L; TiO_2_ loading = 100 mg/L; pH 4.2; Volume: 4 L Temperature: 30 ± 3 °C Reaction time: 90 min UV254 (9 W mercury lamp) 27.8 mW cm^−2^ HC conditions by means of Venturi tube: highest pressure of 0.34 MPa and highest flow rate of 105.7 mL/s.	[46]
TC	US+ Photocatalysis: Reduced Graphene Oxide (rGO)/CdWO_4_ hierarchical structures US-assisted	TC Complete degradation [TC]_0_ = 13.54 mg/L; catalyst loading = 0.216 g/L; pH 5.7; Volume: 500 mL Temperature: 25 ± 2 °C; Reaction time: 60 min: UV.	[47]

SDZ	US + Photocatalysis: MgO/CNT nanocomposites (MCs)	SDZ complete degradation; [SDZ]_0_ = 45 mg/L; [MCs] = 0.9 g/L; pH 11.0; Volume: 200 mL Temperature: room temperature; Reaction time: 80 min; UV intensity: 150 W; US frequency and power: 24 kHz and 200 W/m^2^.	[48]

Levofloxacin (LVO)	Nanophotocatalyst: (BiOBr)_0.75_(Bi_7_O_9_I_3_)_0.25_-U	LVO degradation = 95.4%; [LVO]_0_ = 50 mg/L; Catalyst loading = 1 g/L; pH 6; Volume: 200 mL Reaction time: 120 min; 400 W halogen bulb.	[49]
	US-assisted preparation of catalyst (20 kHz, 200 W)		
TC	Nanophotocatalyst: Bi_2_Sn_2_O_7_-C_3_N_4_/Y zeolite	Highest TC degradation = 80.4%; [TC]_0_ = 20 mg/L; Y Zeolite mass percentage = 10 %; [Bi_2_Sn_2_O_7_-C_3_N_4_/Y zeolite ] = 1 g/L; pH 6; Volume: 200 mL Reaction time: 90 min; Simulated solar light irradiation.	[50]
	US-assisted preparation of catalyst (20 kHz, 150 W)		

**Enzymatic strategies coupled with US/HC**			
Ciprofloxacin (CPFX)	US/Laccase catalyzed degradation	Maximum CPFX degradation = 51%; enzyme loading = 0.02% (*w*/*v*); [CPFX]_0_ = 10 mg/L Volume: 4.5 L Temperature: 60 °C; Reaction time: 6 h (max) US frequency and power: 22 kHz and 75 W.	[51]
**Multiple synergic approaches: comparison**			
AMX, Doxycycline, SDZ	Cavitating jet impingement, UV/Fenton, analogous Fenton, photocatalytic oxidation	COD reduction = 79.9% (maximum obtained COD); Synergetic index 2.125 [H_2_O_2_]_0_ = 500 mg/L; [FeSO_4_]_0_ =10 mg/L; [COD]_0_ = 236 mg/L, pH 2.7; Volume: 50 L Temperature: 49 °C; Reaction time: 60 min UV-light source of 30 W (253.7 nm).	[52]
Oxacillin (OXA)	TiO_2_ photocatalysis, sonochemistry, photo-Fenton process and electrochemistry (with a Ti/IrO_2_anode in sodium chloride).	TiO_2_ photocatalysis: antibiotic removal after 45 min and about 90.0% mineralization after 135 min. Sonochemical system (275 kHz, 60 W): pollutant removed after 120 min. Photo-Fenton process: 100.0% removal and about 35% of the initial TOC after 27 min. Electrochemical oxidation: elimination in 9 min.	[53]

## Acronyms and Abbreviations

AMX	Amoxicillin
AOP	Advanced Oxidation Process
BiOX	Bismuth Oxyhalides
BSO-BCN	Bi_2_Sn_2_O_7_-C_3_N_4_ nano-photocatalyst
CNT	Carbon Nanotubes
COD	Chemical Oxygen Demand
CPFX	Ciprofloxacin
EAOP	Electrochemical Advanced Oxidation Process
HC	Hydrodynamic cavitation
LVO	Levofloxacin
MCs	MgO/CNT nanocomposites
OTC	Oxytetracycline
OXA	Oxacillin
PIE	Pharmaceutical Industry Effluent
PS	Persulfate
rGO	Reduced Graphene Oxide
SDZ	Sulfadiazine
TBA	Tert-Butyl Alcohol
TC	Tetracycline
TOC	Total Organic Carbon
US	Ultrasound or Acoustic cavitation
VLPHS	Visible-Light Responsive Photocatalyst
